# Modular Single-Stage Three-Phase Flyback Differential Inverter for Medium/High-Power Grid Integrated Applications

**DOI:** 10.3390/s22052064

**Published:** 2022-03-07

**Authors:** Ahmed Ismail M. Ali, Cao Anh Tuan, Takaharu Takeshita, Mahmoud A. Sayed, Zuhair Muhammed Alaas

**Affiliations:** 1 Electrical and Mechanical Engineering Department, Nagoya Institute of Technology, Nagoya 466-8555, Japan; t.cao.936@stn.nitech.ac.jp (C.A.T.); take@nitech.ac.jp (T.T.); 2 Electrical Engineering Department, South Valley University, Qena 83523, Egypt; mahmoud_sayed@ieee.org; 3 Electrical Engineering Department, Jazan University, Jazan 45142, Saudi Arabia; zalaas@jazanu.edu.sa

**Keywords:** modular flyback differential inverter (MFBDI), continuous modulation scheme (CMS), static linear strategy (SLS), harmonic compensation strategy

## Abstract

This paper proposes a single-stage three-phase modular flyback differential inverter (MFBDI) for medium/high power solar PV grid-integrated applications. The proposed inverter structure consists of parallel modules of flyback DC-DC converters based on the required power level. The MFBDI offers many features for renewable energy applications, such as reduced components, single-stage power processing, high-power density, voltage-boosting property, improved footprint, flexibility with modular extension capability, and galvanic isolation. The proposed inverter has been modelled, designed, and scaled up to the required application rating. A new mathematical model of the proposed MFBDI is presented and analyzed with a time-varying duty-cycle, wide-range of frequency variation, and power balancing in order to display its grid current harmonic orders for grid-tied applications. In addition, an LPF-based harmonic compensation strategy is used for second-order harmonic component (SOHC) compensation. With the help of the compensation technique, the grid current THD is reduced from 36% to 4.6% by diminishing the SOHC from 51% to 0.8%. Moreover, the SOHC compensation technique eliminates third-order harmonic components from the DC input current. In addition, a 15% parameters mismatch has been applied between the flyback parallel modules to confirm the modular operation of the proposed MFBDI under modules divergence. In addition, SiC MOSFETs are used for inverter switches implementation, which decrease the inverter switching losses at high-switching frequency. The proposed MFBDI is verified by using three flyback parallel modules/phase using PSIM/Simulink software, with a rating of 5 kW, 200 V, and 50 kHz switching frequency, as well as experimental environments.

## 1. Introduction

Recently, the COVID-19 pandemic has disturbed most energy resources and prevented the importation and exportation of fuel between the countries. In addition, the large need for energy in human life has increased the requirements for renewable energy sources (RESs) [1]. Among the different RESs, photovoltaic (PV) is the most common and promising energy resource due to its operation sustainability in the distributed generation systems, which provide freely available energy hunks for humankind [2,3,4]. Therefore, different single-stage and multistage inverter topologies have been developed as an attractive key for grid-integrated RESs at different climatic conditions [5,6,7,8]. In comparison with the multistage inverter, single-stage structures offer reliable, compact, high power density, and improved footprint converters [9,10,11,12,13]. Different multilevel inverter (MLI) structures are presented for high power-quality stand-alone and grid-integrated PV applications [6,8]. However, large-size line-frequency transformers are required in many topologies that decrease the system power density and increase the required components. Owing to galvanic isolation necessity, high-frequency transformer (HFT)-based inverters have been developed for inverter footprint and efficiency improvement [14,15]. HFT-based inverters start with a two-stage operation using a decoupling capacitor/inductor, which increases the required components number, size, and system overall footprint [16,17,18,19].

Motivated by the preceding drawbacks of the two-stage inverter, single-stage inverter architectures have been presented in single/three-phase applications with continuous DC-input current waveform [6]. In [6], single-phase single-stage isolated DC-AC MLI is presented for stand-alone and grid-integrated PV applications. Despite the improved power quality of these converters, many power switches are required. Therefore, PV-microinverters have been widely recommended in recent decades due to their features such as low cost, compactness, reliability, improved footprint, and galvanic isolation requirements for grid-connected applications [20,21]. Microinverter-based flyback DC-DC modules are commonly utilized in combined with the unfolding circuit due to the reduced passive components, a low number of switches, and a simple control strategy [22,23]. However, the recently available PV-microinverters are applicable for low-power purposes (below 200 W). Therefore, DC-DC converter-based differential inverter topologies have been presented for isolated single-stage high power applications. In [24,25], single-phase differential inverters have been introduced with buck and boost converter modules for compact and efficient operation. However, the presented topologies suffer from the high voltage stress over the converter components [26]. In [27], the Cuk differential inverter has been presented for single-phase applications; however, it requires a comparatively increased number of components. Therefore, a single-phase single-stage Cuk differential inverter is proposed for PV direct power conversion with compact and high-efficiency operation [28,29]. However, the proposed control strategy increases the ripples of the DC input current that decreases the input power factor. For three-phase purposes, a three-phase single-stage Cuk differential inverter is proposed for PV applications [30,31]. However, three separate input filters are required for continuous input current operation for RESs. In [32], the three-phase single-stage SEPIC differential inverter is presented with only four switches. However, the presented topology is applicable for low-power applications due to the drop of grid isolation.

In comparison with the former buck-boost based differential inverter topologies, the proposed modular flyback inverter structure has the following merits; it utilizes a reduced number of passive elements and power switches, single-stage DC-AC conversion, voltage boosting, modularity, compact size, and galvanic isolation property. The three-phase flyback differential inverter (FBDI) parameters design and selection, hardware implementation, and control technique were analyzed. Modular FBDI (MFBDI) was presented and analyzed for megawatt-class DC-DC converter-based inverters. Mathematical modelling of the proposed MFBDI was analyzed, which confirmed the existence of a second-order harmonic component (SOHC). In addition, SOHC compensation was achieved by considering an LPF-based harmonic elimination strategy for grid current THD reduction in order to follow the IEC61000-3-2 (Class-A) harmonic standard limit. In order to confirm the modular operation of the proposed MFBDI, a 15% mismatch between the paralleled flyback DC-DC modules was used with three-paralleled modules in each phase for an overall rating of 5 kW. It is worth mentioning that the proposed MFBDI was compared with its recent counterpart topologies, by considering the number of components and passive elements, modulation scheme, number of control loops, controller, switching frequency, current THD, number of required sensors, power rating, and the utilized switches ratings as depicted in Table 1. Obviously, the proposed converter utilizes a reduced number of components, control loops, and sensors that enhances the system footprint and cost.

The paper manuscript has been organized as follows: Section 2 illustrates the modular operation of the proposed MFBDI and its mathematical model. In addition, the flyback converter parameters’ design and selection, as well as hardware implementation, are analyzed in Section 3. Moreover, the MFBDI control scheme and the system simulation and experimental verifications are illustrated and analyzed in Section 4 and Section 5, respectively. Finally, Section 6 concludes the paper’s contributions and verifications.

## 2. Modular Flyback Differential Inverter

**Figure 1:**
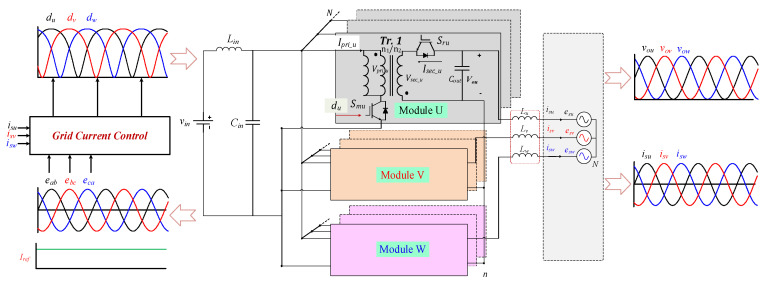
Circuit configuration of the single-stage three-phase MFBDI.

**Figure 2:**
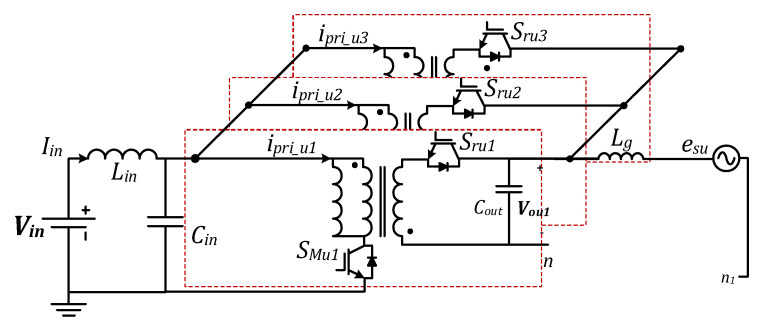
One phase of the proposed MFBDI, N = 3.

The proposed MFBDI consists of N number of isolated DC-DC flyback modules, which are connected in parallel at the input and output sides, Input Parallel Output Parallel (IPOP), as depicted in Figure 1. However, the three-phase configuration is connected in parallel at the DC input side and differentially on the grid side. In addition, the power rating of the MFBDI is simply N-multiple of single module power rating, where N is a number of parallel modules in each phase as shown in Figure 1 and Figure 2. Hence, the operation principles of a single flyback module and mathematical modeling of the proposed MFBDI are analyzed in this section.

### 2.1. Single Flyback Module Operation

**Figure 3:**
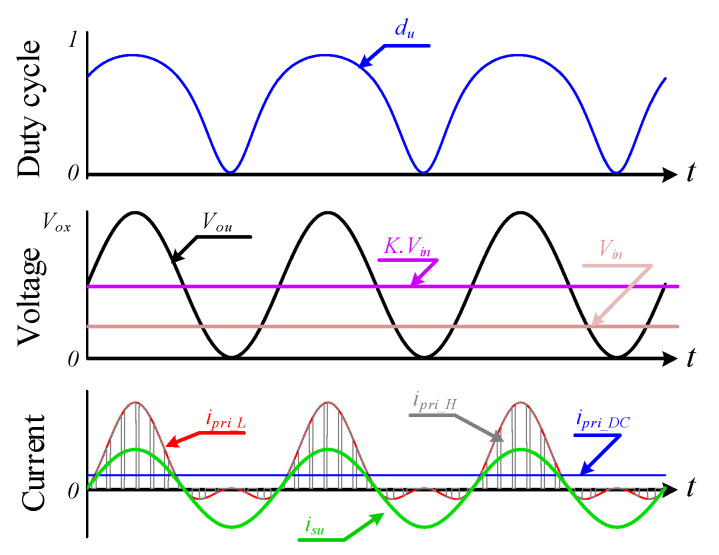
Control waveforms and current/voltage components.

**Figure 4:**
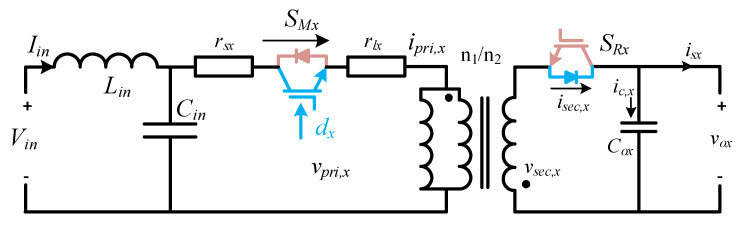
Single module bi-directional power flow of the proposed MFBDI.

**Figure 5:**
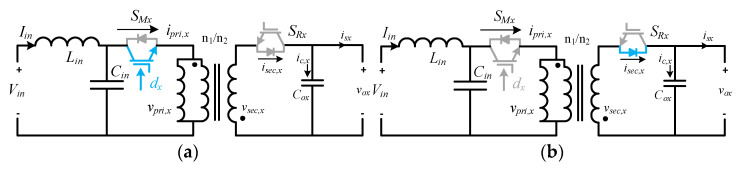
Temporarily power transfer of the MFBDI: (**a**) Energy storage in the HFT magnetizing inductance, (**b**) Energy release to supply the load and charging the output capacitor.

Figure 1 shows the power stage of the proposed MFBDI, where the power stage of one phase of the proposed MFBDI is portrayed in Figure 2. A one-phase of the proposed MFBDI is consists of three parallel modules to increase the system power rating to triple-level (N = 3). A single module is composed of; a single LC input low-pass filter (*L_in_*, *C_in_*), two SiC-MOSFET switches (*S_Mx_*, and *S_Rx_*), a flyback high-frequency transformer (FB-HFT), a robust designed snubber-circuit, and a single output capacitor (*C_Ox_*). The proposed inverter is controlled with a time-varying duty-cycle (*d_x_*). Thus, the generated output voltage at the inverter terminal is DC-voltage with a sinusoidal envelope as depicted in Figure 3. Obviously, the MFBDI terminal voltage contains a DC voltage offset due to the unipolar operation of the flyback modules, which is canceled at the grid side by a differential connection. Hence, a sinusoidal output voltage can be synthesized for sinusoidal grid injected current. In addition, the phase-shift between the flyback modules of the same phase is 0° and 120° between the DC-DC modules of different phases. In addition, a single-module operation can be divided into two operational modes [36]: a. Turing-ON of primary switch passes the current in the primary side of FB-HFT, which stores energy in the HFT magnetic inductance as a magnetic field. The grid-current is maintained by the inverter terminal capacitor during this mode. b. As the main switch turns-OFF, the stored energy releases through the body-diode of the synchronous switch to supply the grid-current and charge the output capacitor as a Current Source Inverter (CSI). The operational modes of a one-phase/single module of the proposed three-phase MFBDI with its bidirectional power flow are portrayed in Figure 4. Moreover, the flyback converter operates temporarily to transfer the power from input to output sides, as clearly portrayed in Figure 5a,b.

The input DC-voltage/PV voltage is assumed to be constant due to LC filter’s large capacitance at the input side. The proposed MFBDI is controlled via continuous conduction-mode (CCM), in which the transformer core incompletely demagnetizes in one switching cycle. The demagnetizing effect of the MFBDI transformer has a negligible effect on the inverter output power-factor as the magnetizing energy is very low compared with the transferred energy to the output side [36]. In addition, the voltage spikes are limited by a stringently designed snubber-circuit.

### 2.2. Mathematical Model of MFBDI

Generally; as the proposed MFBDI is supplied with DC-voltage at the input-side and controlled by time-varying duty-cycle, an output-voltage with sinusoidal envelope can be synthesized at the terminal of each module considering the converter conversion ratio as follows:

(1) $$M\left(d\right)=\frac{nd_{x}}{d_{x}{}^{\prime }}\cdot \left(1-\frac{d_{x}{}^{\prime }\cdot V_{d}}{n\cdot d_{x}\cdot V_{in}}\right)\times \left(\frac{1}{1+\frac{r_{lx}d_{x}n^{2}}{R_{eq}\cdot d_{x}{}^{\prime 2}}+\frac{r_{sx}d_{x}n^{2}}{R_{eq}\cdot d_{x}{}^{\prime 2}}+\frac{r_{d}}{R_{eq}\cdot d_{x}{}^{\prime }}}\right)$$

where,

*M(d) is the input-to-output voltage conversion ratio*,

*V_in_ is the input DC voltage*,

*n is the transformer turns ratio, n = n*_2_*/n*_1_,

*d_x_ is the main switch duty cycle*,

*V_d_ is the voltage drop over the diode*,

*r_lx_ is the primary inductor resistance*,

*R_eq_ is the grid equivalent resistance*,

*r_sx_ is the MOSFET semiconductor switch on-resistance*,

*r_d_ is the diode on-resistance*.

Based on Equation (1), the resistances of the inductor, switches, and diodes limit the MFBDI voltage gain. Thus, maintaining these resistances at a low level is an important aspect [37]. An important issue of the buck-boost-based inverter is the nonlinear relation between the converter input and output, which results in the low-order harmonics in the grid injected currents. In addition, it results in high voltage stress over power components that increases the inverter power loss and diminishes its efficiency. The voltage/current stress over the proposed MFBDI, related to its parameters, is listed in Table 2. Thus, the selection of the different elements rating can be decided to avoid components failure and unstable running operation due to the presence of the passive elements. The passive-elements values determine the system stability over the wide range of duty-cycle and frequency variations.

**Figure 6:**
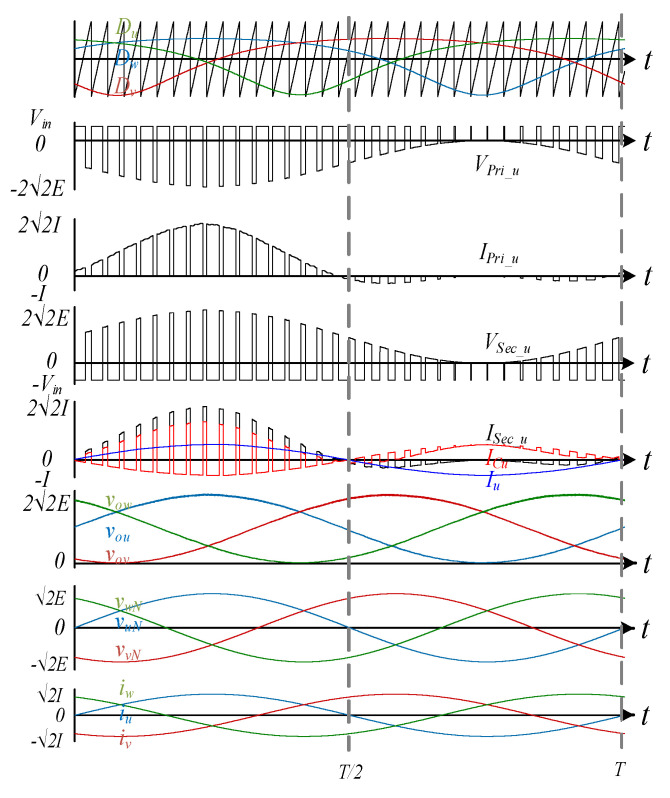
Switching waveforms of single flyback module of the proposed MFBDI.

The switching waveforms of a single flyback module is depicted in Figure 6, which portrays the three-phase duty-cycles (*d_x_*), HFT primary switched voltage and current waveforms (*v_pri_x_*, *i_pri_x_*), switched voltage and current waveforms of HFT secondary side (*v_sec_x_*, *i_sec_x_*), capacitor current *i_cx_*, three-phase output voltages (*v_ox_*), three-phase grid-voltages (*v_x_*), and three-phase grid-injected currents (*i_sx_*). For ideal operation of the proposed three-phase inverter, the three-phase balanced voltages and grid currents can be formulated as follows:

(2) $$\left[\begin{array}{c} v_{su}\left(t\right)\\ v_{sv}\left(t\right)\\ v_{sw}\left(t\right) \end{array}\right]=\sqrt{2}\cdot E\cdot \left[\begin{array}{c} sin\left(\omega t+\alpha \right)\\ sin\left(\omega t+\alpha -\frac{2\pi }{3}\right)\\ sin\left(\omega t+\alpha +\frac{2\pi }{3}\right) \end{array}\right]$$

(3) $$\left[\begin{array}{c} i_{su}\left(t\right)\\ i_{sv}\left(t\right)\\ i_{sw}\left(t\right) \end{array}\right]=\sqrt{2}\cdot I\cdot \left[\begin{array}{c} sin\left(\omega t+\alpha \right)\\ sin\left(\omega t+\alpha -\frac{2\pi }{3}\right)\\ sin\left(\omega t+\alpha +\frac{2\pi }{3}\right) \end{array}\right]$$

where *E* and *I* are the RMS values of the grid voltage and current, respectively. In addition, ω is the grid angular-frequency, and α is the arbitrary angle. 

Due to the parallel connection of flyback modules sharing identical amount of power, then the total grid current can be formulated as follows:

(4) $$\left[\begin{array}{c} i_{su}\left(t\right)\\ i_{sv}\left(t\right)\\ i_{sw}\left(t\right) \end{array}\right]=\sqrt{2}\cdot I\cdot N\cdot \left[\begin{array}{c} sin\left(\omega t+\alpha \right)\\ sin\left(\omega t+\alpha -\frac{2\pi }{3}\right)\\ sin\left(\omega t+\alpha +\frac{2\pi }{3}\right) \end{array}\right]$$

where *N* is the number of parallel modules in each phase of MFBDI.

Based on Equation (1) for the output-to-input voltage transfer ratio of the buck-boost based inverters, the ideal voltage transfer ratio can be formulated as follows:

(5) $$\frac{v_{ox}}{v_{in}}=\frac{i_{in,Nx}}{i_{sxN}}=\frac{d_{x}}{1-d_{x}}$$

where *i_in_*_,*Nx*_, *i_sxN_* are the input and grid currents of module *N* in phase *x*.

Therefore, the duty cycle can be expressed as follows:

(6) $$d_{x}=\frac{v_{ox}}{v_{ox}+v_{in}}$$

Evidently, the buck-boost converter input-to-output nonlinear relation results in low-frequency odd harmonics; however, the low-order even harmonics result from the modules mismatch. Therefore, the proposed MFBDI is controlled in continuous conduction-mode (CCM), in CMS merged with static linearization-strategy (SLS) for low-order odd harmonics minimization. In addition, SOHC is created by the flyback modules mismatch: hence, a secondary loop is required for SOHC elimination. With the help of the properly controlled duty cycle and the DC-DC flyback converter-based operation, the inverter synthesized output voltage has two components; DC offset voltage and line-frequency AC voltage for grid integration. The DC offset voltage is approximately equal for all modules. Thus, the output voltage can be formulated as follows:

(7) $$\left[\begin{array}{c} v_{ou}\left(t\right)\\ v_{ov}\left(t\right)\\ v_{ow}\left(t\right) \end{array}\right]=V_{dc0}+v_{sx}\left(t\right)=V_{dc0}+\sqrt{2}\cdot E\cdot \left[\begin{array}{c} sin\left(\omega t+\alpha \right)\\ sin\left(\omega t+\alpha -\frac{2\pi }{3}\right)\\ sin\left(\omega t+\alpha +\frac{2\pi }{3}\right) \end{array}\right]$$

To decrease the voltage stress over the switches, the peak value of AC component is adjusted to be equal to DC component (*V_dc_*_0_). Thus, the inverter terminal voltages can be formulated as follows:

(8) $$v_{ox}\left(t\right)=V_{dc0}+V_{dc0}\cdot K_{x}=Mv_{in}+Mv_{in}\cdot K_{x}\;=Mv_{in}\cdot \left(1+K_{x}\right)$$

where *M* is the flyback converter voltage-gain and *K* is the instantaneous unity of three-phase waveforms:

(9) $$K_{x}=\left[\begin{array}{c} sin\left(\omega t+\alpha \right)\\ sin\left(\omega t+\alpha -\frac{2\pi }{3}\right)\\ sin\left(\omega t+\alpha +\frac{2\pi }{3}\right) \end{array}\right]$$

From (6) and (8), the static-linearized duty-cycle can be synthesized as follows:

(10) $$d_{x}=\frac{M(1+K_{x})}{M(1+K_{x})+1}$$

The maximum voltage-gain of the flyback module, at *K* = 1, can be formulated as:

(11) $$M=\frac{V_{ox}}{2v_{in}}$$

where *V_ox_* is the peak value of converter terminal voltage. 

Therefore, each flyback module operates with variable duty-cycle to transfer its rated power based on the required voltage transfer ration, which inspire its modular operation by increasing the number of parallel operating modules in each phase of the proposed MFBDI. Hence, the total input current to one phase (*u*) is the sum of all currents to each individual module:

(12) $$i_{in,u}=\overset{N}{\underset{m=1}{\sum }}i_{in,m}$$

Thus, the total input DC current of the MFBDI can be expressed as follows:

(13) $$i_{dc}=i_{in,u}+i_{in,v}+i_{in,w}$$

Based on (5) and (10), the output current of one-phase (*u*) can be formulated as follows:

(14) $$i_{in,Nx}=0.5Mi_{sxN}+MK\cdot i_{sxN}-0.5MK_{1}\cdot i_{sxN}$$

where *K*_1_ is the second-order sinusoidal constant of the inverter input current, which illustrates the circulating power between the different flyback modules at double of the line-frequency as shown in Figure 3. It can be formulated as follows:

(15) $$K_{1}=\left[\begin{array}{c} Cos\left(2\omega t+\alpha \right)\\ Cos\left(2\omega t+\alpha -\frac{2\pi }{3}\right)\\ Cos\left(2\omega t+\alpha +\frac{2\pi }{3}\right) \end{array}\right]$$

Therefore, the input power to a single flyback module can be expressed as follows:

(16) $$p_{in,Nx}=0.5Mv_{in}i_{sxN}+MK\cdot v_{in}i_{sxN}-0.5MK_{1}\cdot v_{in}i_{sxN}$$

## 3. Converter Parameters Design and Selection

**Figure 7:**
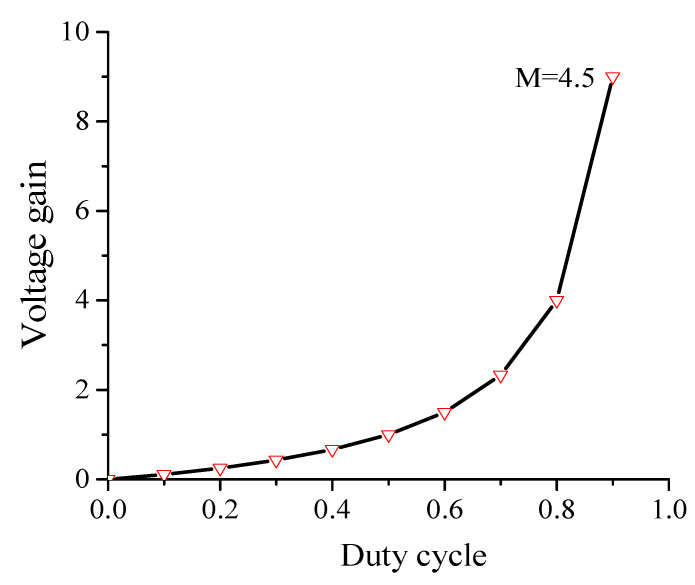
Flyback converter voltage gain.

**Figure 8:**
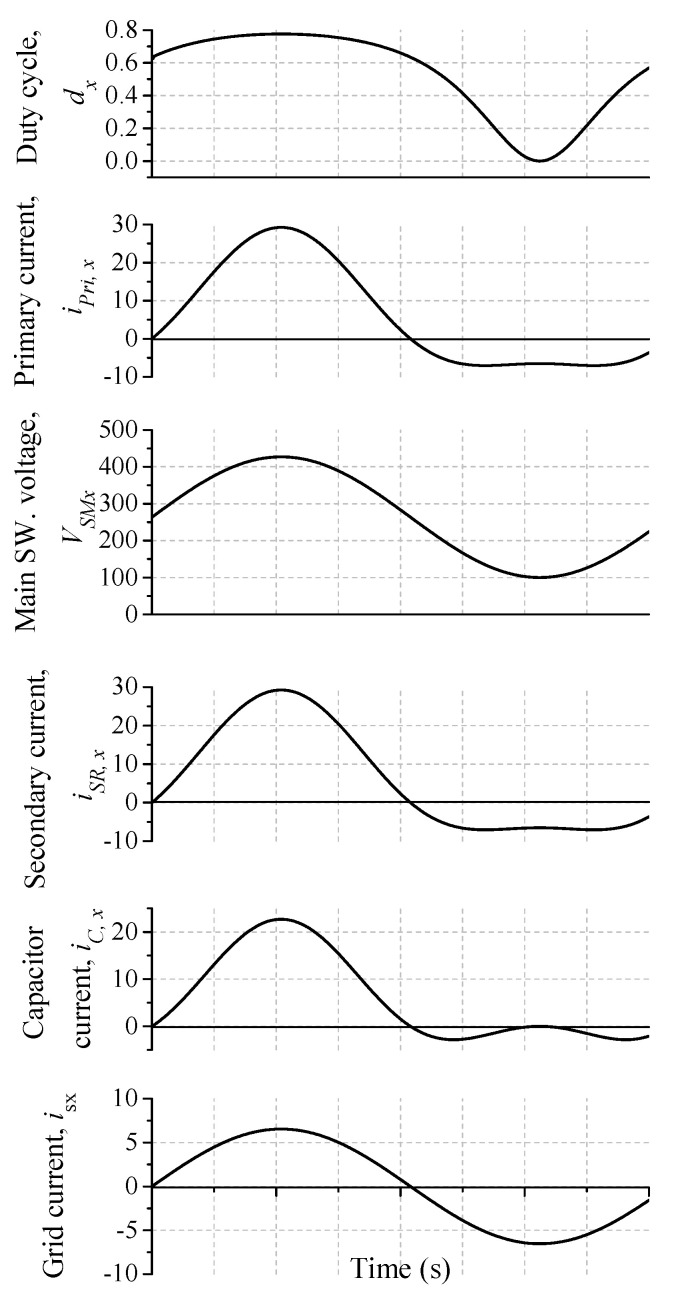
Averaged one-cycle of the proposed MFBDI.

In the parameter selection of the proposed converter, each DC-DC flyback module is designed with reduced switching components. In addition, the designed parameters for the flyback module are listed in Table 3. The plot of flyback converter voltage gain with different duty cycles (*d_x_* = 0~0.9), based on (5) and (11), is seen in Figure 7. The main and synchronous switches of each flyback module (*S_Ma_*, *S_Ra_*) are complementary controlled. In addition, continuous DC input current is an important aspect for renewable energy applications, such as photovoltaics and fuel cells. Therefore, the high-frequency switched differential structure of the proposed converter, together with a single LC input filter, provides the following features: (a) it provides DC input current considering single input filter, (b) it eradicates the large electrolytic capacitor over the input PV modules, (c) it provides galvanic isolation that minimizes the EMI and CMV, and (d) it minimizes the inverter size due to the single-stage operation [30,37]. The large-signal model is used to illustrate the low-frequency component of the current and voltage waveforms to illustrate the components voltage and current stresses. Figure 8 shows the duty cycle, primary current, main switch voltage, synchronous switch current, capacitor average current, and grid-injected current. The flyback module parameters design and selection are cleared in the following sections.

Based on the system parameters that are listed in Table 3, each three-phase module of the MFBDI processes 1.6 kW. Therefore, each flyback module processes one-third of the converter power as follows:

(17) $$P_{Module}=\frac{P_{FBDI}}{3}=\frac{1600}{3}=533.3\;W$$

Thus, each module processes 533.3 W from the DC side to the grid side.

In addition, based on (11) the maximum voltage gain of each flyback converter can be calculated as follows:

(18) $$M=\frac{v_{ox}}{2v_{in}}=\frac{2\times 163.3}{2\times 100}=1.633$$

Therefore, the minimum and optimal value for *M* is (1.633) for inverter differential operation.

Moreover, the required grid-injected current can be calculated as follows:

(19) $$P_{Module}=E\cdot i_{x}=533.3\;W\;i_{x}=4.6185\;A$$

where *i_x_* refers to the RMS value of the grid injected current.

Considering the maximum operating duty cycle (*d_x_* = 0.8) during the converter parameters design, the peak value of input current to the converter can be calculated as follows:

(20) $$I_{in}=\left[\frac{d_{x}}{1-d_{x}}+1\right]\cdot I_{x}=32.66\;A$$

Consequently, the grid equivalent resistance is 25 Ω and the voltage and current stresses can be calculated as follows:

(21) $$V_{SMx}=v_{in}+v_{ox}=100+\frac{2\times 200\sqrt{2}}{\sqrt{3}}=426.5\;V\;I_{SMx}=32.66\;A$$

### 3.1. Passive Elements Design

Based on (6), the proposed inverter operating at a wide-range of duty cycle variation, which disturbs the converter dynamic and its stability. Therefore, a robust design of converter passive components is very important aspect. The acceptable limits of the capacitor voltage and inductor current ripples are less than 10% and 20%, respectively [37]. In addition, 50 kHz switching frequency is selected for ripples minimization and passive elements size reduction.

According to the former permissible limits for the output voltage and current ripples, the HFT required magnetizing inductance can be designed as follows [37,38]:

(22) $$L_{Mx}=\frac{D_{x}\times V_{in}}{2\times \Delta I\times F_{SW}}\;=\frac{0.8\times 100}{2\times 40\times 0.2\times 50000}=100\;\mu H$$

Moreover, the converter output capacitor can be designed as follows:

(23) $$C_{ox}={\left(\frac{d_{x}}{1-d_{x}}\right)}^{2}\times \frac{V_{in}}{2\times R_{eq}\times \Delta V\times F_{SW}}\;={\left(\frac{0.8}{1-0.8}\right)}^{2}*\frac{100}{2*25*0.1*500*50000}=12.8\;\mu F$$

Therefore, the HFT magnetizing inductance and output capacitor of each flyback converter module are selected as 100 µH and 12.8 µF, respectively.

### 3.2. Hardware Implementation

Different outstanding magnetic materials are utilized for high frequency transformers/inductors design, such as monocrystalline, ferrite, nanocrystalline, amorphous, and powder magnetic materials. A comparison between these materials has been demonstrated in [38,39]. Soft ferrite materials are the common, low cost, and acceptable power loss in low power applications for micro-inverters power-level applications [39]. Thus, EER-94 soft ferrite core is used for the proposed converter module implementation. The core parameters are: *A_e_* = 712 mm^2^ effective cross-section area, *V_e_* = 158,000 mm^3^ effective volume, and permeability *µ* = 2500. In addition, LITZ wire has been used for HFT implementation for eddy current minimization to improve the system efficiency.

Recent isolated converters utilize a complicated magnetic structure that is composed of multiple heterogeneous elements (core material + air gap) and different winding layers. The terminal voltage of the proposed MFBDI is variable due to the variable duty cycle; therefore, the number of turns can be obtained as follows [38,39];

(24) $$N=\frac{V_{pri\_x\left(max\right)}}{K_{v}\times B\times A_{e}\times F_{SW}}$$

where,

*V_pri_x_*_(*max*)_*is the maximum primary voltage of the HFT*,

*K_v_ is waveform factor*,

*B is the flux density*,

*A_e_ the core cross section area*.

Moreover, the length of the required air gap is set to 1.6 mm for rated power operation, which can be formulated as follows [39]:

(25) $$l_{e}=\frac{N^{2}\cdot \mu_{o}\cdot A_{Core}}{L_{m}}$$

where *µ_o_* is the free space permeability and *l_e_* is the air-gap length.

**Figure 9:**
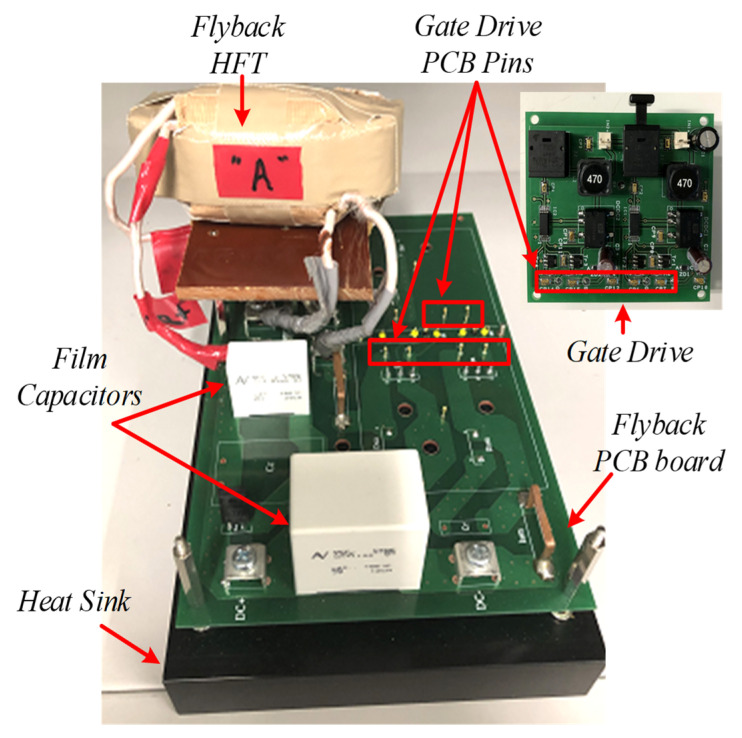
PCB board of a flyback converter module.

Based on (24), the required number of turns for EER soft-ferrite core is 10, which is increased to 15 to maintain the required inductance when the air gap is included. Figure 9 show the PCB board of a single flyback module with the designed elements and power switches.

### 3.3. Input LC Filter Design

A single second-order LC low pass filter is used at the inverter input side for continuous DC input current with input ripples alleviation for renewable energy applications. The input filter is designed at a resonating frequency of *f_o_*, which can be expressed as [37];

(26) $$f_{o}=\frac{1}{2\pi \cdot \sqrt{L_{in}C_{in}}}$$

where the resonating frequency must follow the following expression:

(27) $$10f_{g}\leq f_{o}\leq \frac{f_{sw}}{10}$$

Therefore, *f_o_* is selected as 4 kHz in which the input filter inductance and capacitance are 150 µH and 10 µF, respectively.

## 4. MFBDI Control Scheme

As earlier discussed, the flyback modules are controlled over a wide-range of duty-cycle variation to shape the modulus of sinusoidal voltage waveform with 120° phase-shift between the modules of different phases. Based on (2) and (3), assuming lossless operation of the proposed inverter, and by applying power balance of each phase of MFBDI, the instantaneous grid-powers (*p_u_*, *p_v_*, and *p_w_*) can be expressed as follows:

(28) $$p_{u}=2E_{su}i_{su}{sin}^{2}\left(\omega t+\alpha \right)$$

(29) $$p_{v}=2E_{sv}i_{sv}{sin}^{2}\left(\omega t+\alpha -\frac{2\pi }{3}\right)$$

(30) $$p_{w}=2E_{sw}i_{sw}{sin}^{2}\left(\omega t+\alpha +\frac{2\pi }{3}\right)$$

Moreover, the input power to each module can be expressed as follows:

(31) $$p_{x}=v_{in}i_{in,mx}\left(t\right)$$

where *m* = (1, …, *N*), *x* = (*u*, *v*, *w*)

By applying power balance, therefore, the input currents can be formulated as follows:

(32) $$i_{mu}\left(t\right)=\frac{2E_{su}i_{su}}{v_{in}}{sin}^{2}\left(\omega t+\alpha \right)$$

(33) $$i_{mv}\left(t\right)=\frac{2E_{sv}i_{sv}}{v_{in}}{sin}^{2}\left(\omega t+\alpha -\frac{2\pi }{3}\right)$$

(34) $$i_{mw}\left(t\right)=\frac{2E_{sw}i_{sw}}{v_{in}}{sin}^{2}\left(\omega t+\alpha +\frac{2\pi }{3}\right)$$

where (*E_su_*, *E_sv_*, *E_sw_*) and (*i_su_*, *i_sv_*, *i_sw_*) are the grid RMS voltages and currents, respectively.

Based on the former equations, the input current for each module has a squared sinusoidal waveform due to the virtual unfolding stage offered by the differential connection at the grid side. Thus, the reference input currents for the proposed MFBDI can be formulated as follows:

(35) $$i^{*}{}_{mu}\left(t\right)=\frac{2E_{su}}{v_{in}}I_{su}{sin}^{2}\left(\omega t+\alpha \right)$$

(36) $$i^{*}{}_{mv}=\frac{2E_{sv}}{v_{in}}I_{sv}{sin}^{2}\left(\omega t+\alpha -\frac{2\pi }{3}\right)$$

(37) $$i^{*}{}_{mw}=\frac{2E_{sw}}{v_{in}}I_{sw}{sin}^{2}\left(\omega t+\alpha +\frac{2\pi }{3}\right)$$

where *I_su_*, *I_sv_*, and *I_sw_* are peak values of grid-injected currents that can be decided by the PV MPPT technique in solar PV applications. In addition, the constant in these equations is used as

(38) $$H_{x}=\frac{2E_{sx}}{v_{in}}I_{sx}$$

Based on the control-to-output transfer function of the flyback converter, the flyback module has a right half-plane zero (RHPZ) that affects the inverter stability during the inverter operation over a wide-range of duty-cycle variation [37]. Therefore, an accurate dynamic model is very important. Thus, the control-to-output transfer function of the flyback module can be expressed as follows [34,37];

(39) $$G_{vdx}\left(s\right)=\frac{v_{ox}\left(s\right)}{d_{x}\left(s\right)}=G_{0}\cdot \frac{b_{0}+b_{1}S+b_{2}S^{2}+b_{3}S^{3}}{a_{0}+a_{1}S+a_{2}S^{2}+a_{3}S^{3}}$$

where *G*_0_ is the DC gain, (*b*_0_–*b*_3_) are constants decides the zero locations, and (*a*_1_–*a*_3_) are the constants of poles locations, which are formulated in Table 4 based on the system parameters.

Therefore, a robust designed compensator is required to maintain the inverter stability over a wide-range of duty-cycle and frequency variations. Considering the order of the proposed MFBDI transfer function, a Type-II compensator is used to stabilize the system, where its transfer function can be expressed as follows [40]:

(40) $$G_{cx}\left(s\right)=G_{c0}\cdot \frac{\left(1+\frac{S}{\omega_{z1}}\right)}{\left(1+\frac{S}{\omega_{p1}}\right)\cdot \left(1+\frac{S}{\omega_{p2}}\right)}$$

where $G_{c0}$ is compensator open-loop gain,

$\omega_{z1},\;\omega_{p1},\;\mathrm{and}\;\omega_{p2}$ are frequency locations of converter zeros and poles, respectively.

The compensator static gain, *G_c_*_0_, boosts the DC-gain of the converter open-loop transfer function, which enhances the controller steady state error. In addition, the compensator PID zero (*ω_z_*_1_) improves the flyback converter phase-margin (PM) that enhances the system stability and stretches its bandwidth. Moreover, the two poles of the PID compensator are located, one at low-frequency (*ω_p_*_1_), and the other at high-frequency close to switching frequency (*ω_p_*_2_). The low-frequency pole flattens the converter DC-gain that improves the controller accuracy; however, high-frequency pole eliminates the high-frequency oscillations and damps the converter switching harmonics.

In addition, the mismatch between the flyback modules flows circulating power between converter modules, which results in the SOHC in the grid-injected currents as illustrated in (15) and (16). Therefore, the proposed control strategy comprises two control loops; the main and secondary control loops. The main control loop regulates the grid currents by controlling the converter three-phase primary currents: however, the secondary control loop eliminates the negative sequence SOHC for sinusoidal grid injected currents. Low-pass filter (LPF), triggered at (−2*ω*), is used for SOHC mitigation in the secondary control-loop. The three-phase grid currents are sensed in which SOHCs are extracted using LPFs, and consequently eradicated from the grid currents. The LPF transfer function as be expressed as follows:

(41) $$G_{s}\left(s\right)=\frac{1}{\left(1+\frac{S}{\omega_{n}}\right)}$$

**Figure 10:**
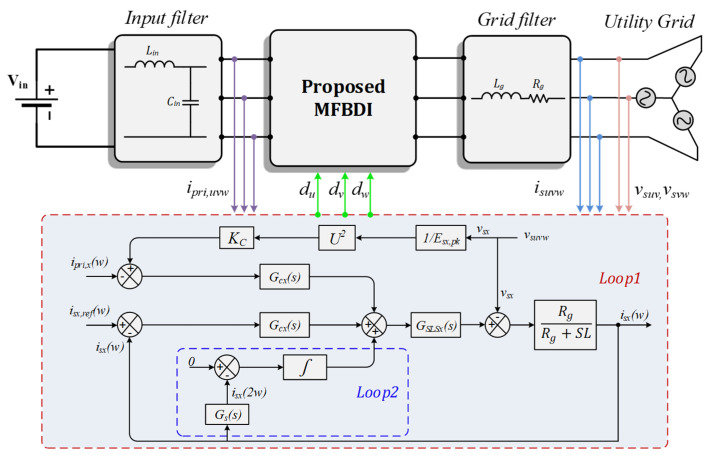
Control block diagram of the proposed MFBDI.

The closed-loop control scheme of the proposed MFBDI for grid-integrated operation is portrayed in Figure 10. Moreover, the overall open-loop transfer function of the converter can be expressed as follows [37]:

(42) $$T\left(s\right)=G_{vdx}\left(s\right)\cdot G_{lgx}\left(s\right)\cdot G_{c1x}\left(s\right)\cdot G_{pwm}\left(s\right)\cdot H_{sx}\left(s\right)\cdot G_{SLS}\left(s\right)$$

where *T(s) is open-loop transfer function*,

*G_vdx_(s) is control-to-output transfer function*,

*G_lgx_(s) is grid filter transfer function*,

*G_c_*_1*x*_*(s) is compensator transfer function considering LPF*,

*G_pwm_(s) is modulator transfer function*, 

*H_sx_(s) is sensor transfer function*, 

*G_SLS_(s) is static linearization strategy of converter duty cycle.*

In addition, the static linearized duty cycle of the proposed flyback module can be expressed, based on (10), as follows:

(43) $$G_{SLSx}\left(s\right)=\frac{m\left(s\right)\cdot \left(1+K_{x}\right)}{m\left(s\right)\cdot \left(1+K_{x}\right)+1}$$

where *m*(*s*) is the compensator’s output signals or static gain of the small-signal component of the differential converter.

**Figure 11:**
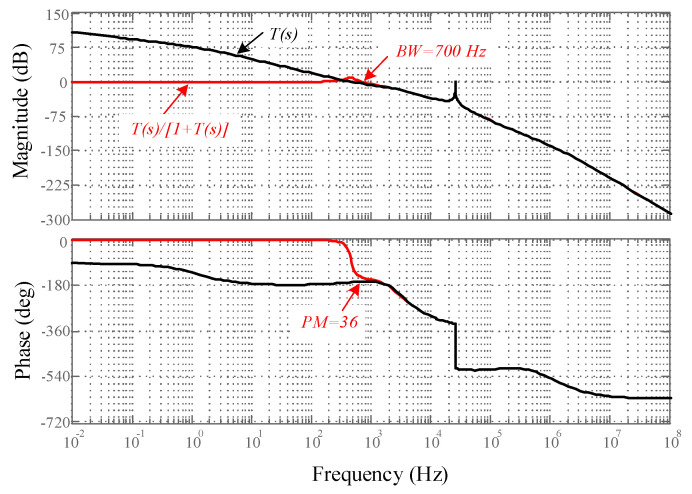
Bode plot of the proposed control scheme of MFBDI.

Moreover, the bode plot of the inverter transfer function, considering the LPF, is depicted in Figure 11 using MATLAB/Simulink software. The proposed compensator stabilizes the MFBDI over the wide-range of duty cycle variations. Considering the proposed control strategy, the converter closed-loop transfer function DC-gain and bandwidth are 91 dB and 700 Hz, respectively. In addition, the converter phase margin is 36 degrees, which confirms converter stability over a wide-range of duty cycle and frequency variations. 

## 5. System Verification

### 5.1. Simulation Results

**Figure 12:**
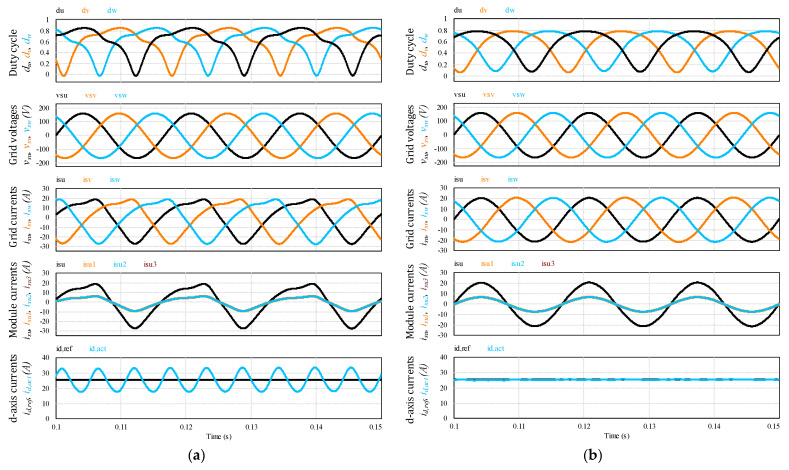
Simulation results of the proposed MFBDI at 5 kW; (**a**) with SOHC, (**b**) without SOHC.

The proposed MFBDI is verified by using PSIM simulation software of 5 kW modular flyback differential inverter architecture considering three paralleled flyback modules in each phase. The simulation parameters of the proposed MFBDI are listed in Table 5. Figure 12 shows the simulation results of the grid integrated MFBDI with and without the SOHC compensation strategy. In both cases, the waveforms of the proposed converter are depicted as follows: duty cycles (*d_u_*, *d_v_*, *d_w_*), grid voltages (*v_su_*, *v_sv_*, *v_sw_*), grid currents (*i_su_*, *i_sv_*, *i_sw_*), u-phase currents (*i_su_*, *i_su_*_1_, *i_su_*_2_, *i_su_*_3_), and d-q axis currents (*i_d_*_,*ref*_, *i_d_*_,*act*_.). The grid injected currents have a high SOHC, which increases the THD of the grid currents. With the proposed LPF-based compensation strategy, the MFBDI supplies the grid with sinusoidal current waveforms with low contained THD. It is worth mentioning that in both cases, the proposed control algorithm manages the power-sharing between the parallel modules for high power applications.

**Figure 13:**
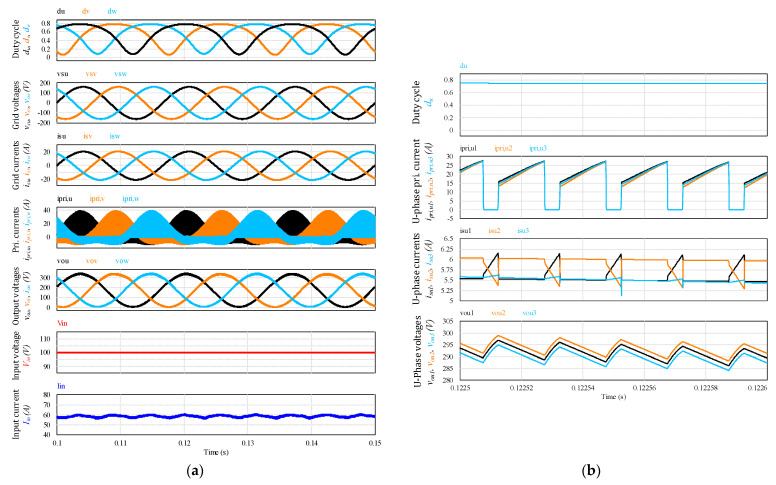
Simulation results of the proposed MFBDI at 5 kW considering 15% parameter mismatch. (**a**) Power frequency waveforms; (**b**) switching frequency waveforms.

In addition, a 15% mismatch between the parameters of the flyback parallel modules of (u-phase) to investigate the proposed converter operation during parameters divergence. The u-phase parameters mismatch is considered according to Table 6. Figure 13 shows the system results considering the parameters mismatch. Figure 13a shows the waveforms of duty cycles, grid voltages, grid currents, primary currents, output voltages, and the input DC voltage and current to confirm the system stable operation during the circuit parameters mismatch. Moreover, the input current ripples are alleviated for continuous input current operation for renewable energy applications. In addition, Figure 13b depicts zoomed region of the system results to illustrate the parameters mismatch effects on the primary currents, grid currents, and output voltages of phase (u). Clearly, the converter has small deviations between the primary currents, grid supplied currents, and output voltages waveforms, which have a negligible effect on the converter overall operation.

### 5.2. Experimental Results

**Figure 14:**
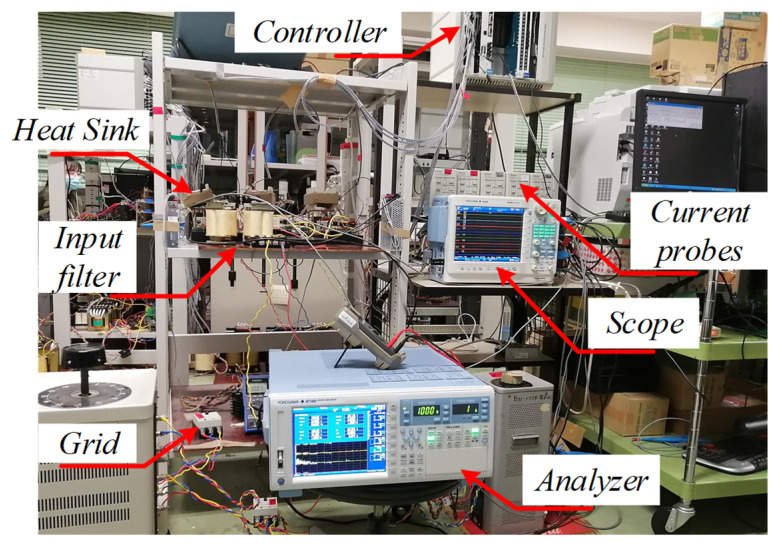
Experimental system prototype photograph.

**Figure 15:**
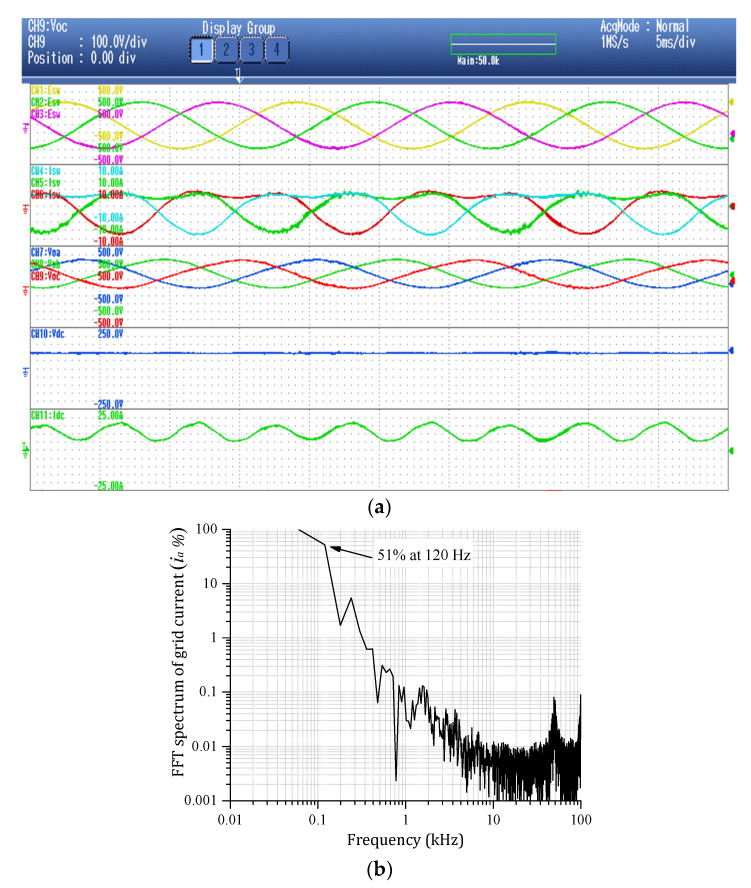
Experimental system results without SOHC compensation. (**a**) Converter results without SOHC compensation. (**b**) Grid current FFT harmonic spectrum without SOHC compensation.

**Figure 16:**
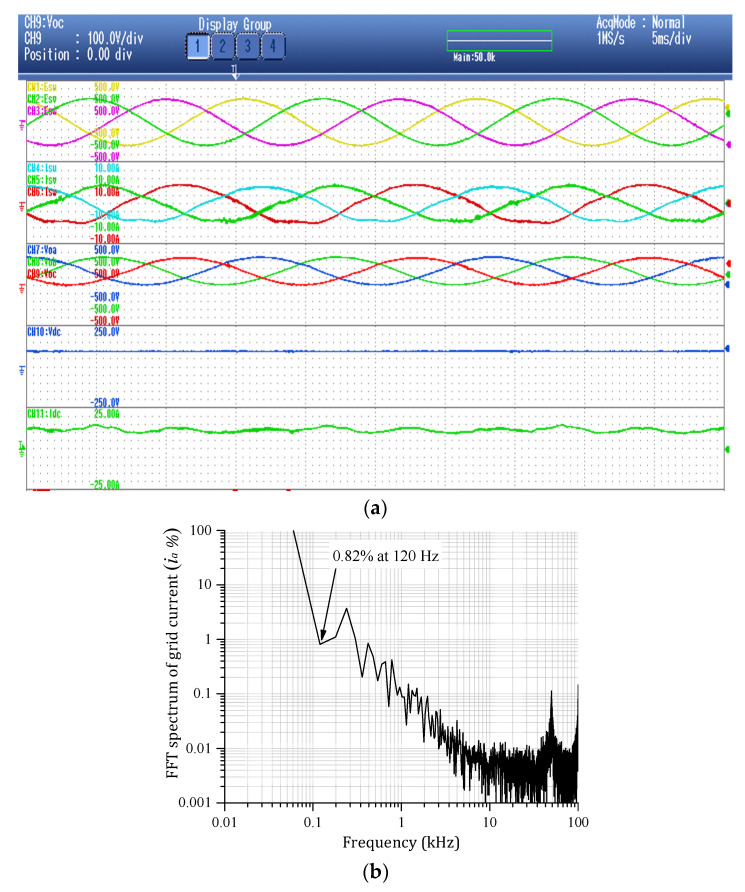
Experimental system results with SOHC compensation. (**a**) Converter results with SOHC compensation. (**b**) Grid current FFT harmonic spectrum with SOHC compensation.

In order to validate the proposed three-phase MFBDI, a single module/phase experimental system prototype was carried out for the proposed DFBDI. Hence, the proposed system was validated via a single module/phase 1.6 W, 200 V, and 50 kHz switching frequency experimental prototype. The system prototype photograph is depicted in Figure 14. The system prototype considers three flyback modules utilizing a single module in each phase. SiC MOSFETs (C2M0040120D) are used for the converter switches in the experimental system, which decrease the system losses and improves the system overall efficiency. In addition, a simple robust designed RC snubber-circuit was designed according to [37] for voltage spikes mitigation due to the existence of the HFT leakage inductance. Moreover, the proposed MFBDI is controlled experimentally using a DSP (TMS320C6713A, TI) digital controller. The proposed system experimental parameters are listed in Table 7. The MFBDI experimental results were captured from the screen of a Yokogawa DL850E digital oscilloscope considering two operating conditions; without and with SOHC compensation. In both cases, the captured experimental waveforms are arranged as follows: three-phase grid voltages, grid currents, output voltages, input DC voltage, and input DC current, respectively. Without SOHC compensation, Figure 15 shows the input and output waveforms of the proposed MFBDI. Clearly, the grid currents are distorted by high SOHC of 51%, which matches the former simulation results, which increases the grid current THD to 36% that exceeds the IEEE and IEC harmonic standard limits. In addition, the SOHC in the grid currents distorts the converter input DC current with a third harmonic order as clearly portrayed in Figure 15, which is important for renewable energy applications such as solar PV and fuel cells. On the other hand, the converter experimental results after the harmonic elimination strategy are depicted Figure 16. With the help of the harmonic compensation strategy, the SOHC was eliminated from the grid-injected currents and the SOHC was reduced to 0.82%, as shown in Figure 16b. Thus, the grid-injected currents are almost sinusoidal waveforms with a THD of 4.6%, which matches IEEE and IEC harmonic standard limits. Moreover, the SOHC compensation strategy eliminates the third order harmonic component from the DC input current, as depicted in Figure 16a, which is important for solar PV applications. Moreover, the single LC input filter mitigates input current ripples, which offers an important feature for renewable energy applications. It worth mentioning that the voltage stress of the converter components is within the designed region due to the robust designed snubber circuit. 

**Figure 17:**
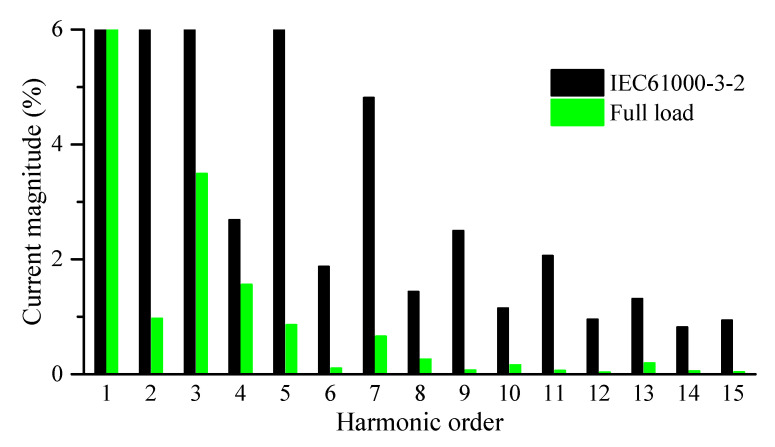
Grid current harmonic orders vs. IEC61000-3-2 (Class-A).

**Figure 18:**
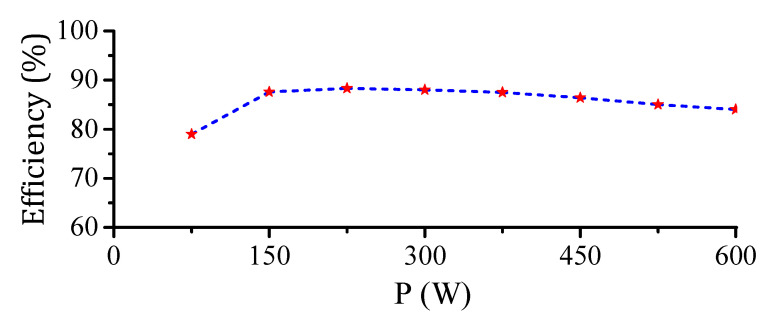
Efficiency profile of a single flyback module of the proposed MFBDI.

In addition, the harmonic orders of the grid injected currents are compared with the IEC 63000-3-2(Class-A) harmonic standard, as depicted in Figure 17. Obviously, the grid current harmonic orders are within the acceptable limit up to the 15th order, which confirms the operation of the SOHC compensation loop in the system control scheme. Finally, the proposed converter efficiency profile was analyzed considering a single flyback DC-DC module operation from the proposed MFBDI between (75 and 600 W) as depicted in Figure 18. Obviously, the converter efficiency is low for low-power operation due to the system conduction loss. However, the system efficiency enhances with increasing the power processed by the proposed converter to reach its optimal value and start to decrease again as depicted in the converter efficiency profile portrayed in Figure 18.

## 6. Conclusions

A modular single-stage three-phase differential inverter is presented in this paper based on the flyback DC-DC module. The proposed converter is verified via three-parallel flyback modules for 5 kW power rating for renewable energy applications. A detailed mathematical model of the proposed MFBDI confirming the SOHC is provided in this paper. Module parameters design strategy are illustrated for acceptable voltage and current ripples. In addition, a new control scheme based-on flyback primary current detection is used to control the grid integrated operation. Two control loops are used in the proposed control scheme for grid currents regulation (loop-1) and SOHC compensation (loop-2), respectively. To ensure the modular operation of the proposed converter architecture, 15% parameters mismatch is applied between the parallel flyback modules to confirm the modular operation of the MFBDI over flyback modules divergence. For experimental validation of the MFBDI, a laboratory prototype based 1.6 W/200 V was built and the experimental results of the proposed converter without and with the LPF-based harmonic elimination strategy are carried out. Clearly, the experimental results follow the simulation results that confirms the robust design of the converter. Moreover, the grid current harmonic orders are compared with the IEC61000-3-2 (Class-A), which are within the standard permissible limit. Furthermore, the converter efficiency profile is analyzed at different power levels. 

## Figures and Tables

**Table 1:** Comparison study for MFBDI with its counterpart topologies.

Control/Ref.	[29]	[33]	[34]	[35]	Proposed
**Switch No.**	*4*	*6*	*6*	*5*	*6*
**Diodes No.**	*4*	*6*	*6*	*4*	*0*
**Inductor No.**	*7*	*9*	*6*	*4*	*1*
**Capacitor No.**	*6*	*9*	*0*	*3*	*4*
**Modulation scheme**	*DMS*	*DMS*	*CMS*	*CMS*	*CMS*
**No. of loops**	*5*	*3*	*3*	*2*	*2*
**Controller**	*PR*	*NA*	*PR*	*Hysteresis*	*PI*
**F_SW_ (kHz)**	*100*	*125*	*25*	*120*	*50*
**THD (%)**	*4*	*4*	*1.2*	*1.9*	*4.5*
**No. of sensors**	*7*	*7*	*7*	*4*	*5*
**Power rating, W**	*500* *(Single-phase)*	*500* *(Three-phase)*	*2500* *(Three-phase)*	*210* *(Single-phase)*	*1600* *(Three-phase)*
Switch rating	*(GS66508P)* *650 V*, *30 A*	*NA*	*(IRG7PH50K10D)* *1200 V*, *90 A*	*FDP51N25* *IPAW60R190CE*	*(C2M0040120D)* *1200 V*, *60 A*

NA: Not Available.

**Table 2:** Voltage and current stresses.

Component	Voltage	Current	Ripple Component
*C_in_*	*v_in_* (*V_PV_*)	$C_{in}\cdot \frac{dv_{in}}{dt}$	$\frac{n\cdot d_{x}\cdot i_{x}}{C_{ox}\cdot f_{sw}}$
*L_Mx_ (HFT)*	$\frac{v_{ox}}{n}$	*n·i_x_*	$\frac{-d_{x}\cdot v_{in}}{L_{mx}\cdot f_{sw}}$
*C_ox_*	*v_ox_*	$i_{x}\cdot \left(\frac{1}{\left(1-d_{x}\right)}-1\right)$	$\frac{d_{x}\cdot i_{x}}{C_{in}\cdot f_{sw}}$
*Primary switch* *(S*_1_ *or S*_2_ *)*	$v_{in}+\frac{v_{ox}}{n}$	$\frac{n\cdot i_{x}}{\left(1-d_{x}\right)}$	$\frac{d_{x}\cdot v_{in}}{L_{mx}\cdot f_{sw}}$
*Secondary switch* *(S*_3_ *or S*_4_ *)*	*v_ox_*	$\frac{i_{x}}{\left(1-d_{x}\right)}$	$\frac{d_{x}\cdot v_{in}}{L_{mx}\cdot f_{sw}}$

where *x* = *u*, *v*, or *w*.

**Table 3:** Flyback converter parameters.

Input DC voltage, *V_dc_*	100 V
Input filter, *L_in_*, *C_in_*	150 µH, 10 µF
Grid voltage (*L.L*), *V_g_*	200 V, 60 Hz
Grid filter, *L_g_*	4 mH
HFT magnetizing inductance, *L_Mx_*	115 µH
HFT leakage inductance, *L_Leakage_*	2.25 µH
Output capacitor, *C_Ox_*	12.8 µF
HFT turns ratio, *n*	1:1
Switching Frequency, *F_SW_*	50 kHz

**Table 4:** Transfer Function Parameters.

$b_{3}=C_{in}DL_{in}{}^{2}L_{M}V_{in}$	$a_{3}=C_{in}L_{in}{\left(1-D\right)}^{2}\left[L_{in}L_{M}+C_{o}R_{eq}\left(L_{in}r_{L}+L_{M}R_{in}\right)\right]$
$b_{2}=-C_{in}DV_{in}\left[\left(L_{in}r_{L}\right)+\left(L_{M}R_{in}\right)+L_{in}R_{eq}\left(2-D\right)\right]$	$\begin{array}{cc} a_{2}=L_{in}{\left(1-D\right)}^{2} & [C_{in}L_{in}R_{eq}{\left(1-D\right)}^{2}+C_{in}\left(L_{in}r_{L}+L_{M}R_{in}\right)\\ & +C_{o}(L_{M}R_{eq}+D^{2}+C_{in}R_{eq}R_{in}r_{L})] \end{array}$
$\begin{array}{r} b_{1}=-V_{in}[DL_{in}\left(DL_{in}+C_{in}R_{in}r_{L}+L_{M}\right)\\ \;-C_{in}L_{M}R_{eq}r_{L}{\left(1-D\right)}^{2}] \end{array}$	$\begin{array}{cc} a_{1}=L_{in}{\left(1-D\right)}^{2} & [L_{M}+D^{2}L_{in}+C_{in}R_{eq}R_{in}{\left(1-D\right)}^{2}\\ & +C_{o}R_{eq}\left(R_{in}D^{2}+r_{L}\right)] \end{array}$
$b_{0}=V_{in}\left[R_{eq}{\left(1-D\right)}^{2}-D\left(DR_{in}+r_{L}\right)\right]$	$a_{0}={\left(1-D\right)}^{2}\left[R_{eq}{\left(1-D\right)}^{2}+r_{L}+R_{in}D^{2})\right]$

where *L_M_* is the HFT magnetizing inductance, *C_o_* or *C_out_* is the flyback module output inductance, *R_eq_* is the grid equivalent resistance, *R_in_* is the resistance of the input inductor, *r_L_* is the resistance of the HFT magnetizing inductor.

**Table 5:** Simulation parameters of the MFBDI.

Rated inverter power, *P*	5 kW
Input DC voltage, *V_dc_*	100 V
Input filter, *L_in_*, *C_in_*	150 µH, 10 µF
Input filter resistance, *r_in_*	2 Ω
Grid voltage *(L.L)*, *E*, *ω*	200 V, 2 × π × 60 rad/s
HFT magnetizing inductance, *L_Mx_*	115 µH
HFT primary resistance, *r_M_*	50 mΩ
Output capacitor, *C_Ox_*	12.8 µF
HFT leakage inductance, *L_Leakage_*	2.25 µH
HFT turns ratio, *n*	1:1
Grid filter, *L_g_*	4 mH
Grid inductor resistance, *r_g_*,	25 mΩ
Switching Frequency, *F_SW_*	50 kHz
PI controller gains, *K_P_*, *K_I_*	0.097 A/V, 280 rad/s

**Table 6:** Parameter mismatch of the proposed MFBDI.

Element	Divergence Values	Average Current/Voltage	Mismatch Percentage
**HFT Magnetizing inductance**	L_M_(u1) = 115 µH	21 A	3.57% (0.75 A)
	L_M_(u2) = 132.25 µH	21.75 A	
	L_M_(u3) = 97.75 µH	20.25 A	
**HFT Leakage inductance**	L_LK_(u1) = 2 µH	21 A	3.57% (0.75 A)
	L_LK_(u2) = 2.3 µH	21.75 A	
	L_LK_(u3) = 1.7 µH	20.25 A	
**Output capacitor**	C_o_(u1) = 12.8 µH	163.3 V	0.6124% (1 V)
	C_o_(u2) = 14.72 µH	164.3 V	
	C_o_(u3) = 10.88 µH	162.3 V	

**Table 7:** Experimental parameters of the MFBDI.

Rated inverter power, *P*	1650 kW
Input DC voltage, *V_dc_*	100 V
Input filter, *L_in_*, *C_in_*	152.3 µH, 10 µF
Input filter resistance, *r_in_*	1.5 Ω
Grid voltage (*L.L*), *E*, *ω*	200 V, 2 × π × 60 rad/s
HFT magnetizing inductance, *L_Mx_*	115.52 µH
HFT primary resistance, *r_m_*	50 mΩ
Output capacitor, *C_Ox_*	12 µF
HFT leakage inductance, *L_Leakage_*	2.56 µH
HFT turns ratio, *n*	1:1
Grid filter, *L_g_*	4 mH
Grid inductor resistance, *r_g_*,	25 mΩ
Switching Frequency, *F_SW_*	50 kHz
PI controller gains, *K_P_*, *K_I_*	0.097 A/V, 280 rad/s

## Data Availability

Not applicable.
